# Hair safe study: Effects of scalp cooling on hair preservation and hair regrowth in breast cancer patients receiving chemotherapy - A prospective interventional study

**DOI:** 10.1016/j.breast.2022.04.008

**Published:** 2022-04-30

**Authors:** Christine Brunner, Miriam Emmelheinz, Ricarda Kofler, Samira Abdel Azim, Marlene Lehmann, Verena Wieser, Magdalena Ritter, Anne Oberguggenberger, Christian Marth, Daniel Egle

**Affiliations:** a Department of Obstetrics and Gynecology, Medical University of Innsbruck, Innsbruck, Austria; b Psychosomatics and Medical Psychology- Psychiatry II, Medical University of Innsbruck, Innsbruck, Austria

**Keywords:** Breast cancer, Chemotherapy, Alopecia, Scalp cooling, Hair loss, Regrowth

## Abstract

• Scalp cooling significantly reduced chemotherapy-induced-alopecia in breast cancer patients.
• No significant effect regarding regrowth after chemotherapy in scalp cooling group.
• Scalp cooling is more effective in preventing alopecia in patients receiving taxane monotherapy.

## 1 Introduction

Breast cancer is the most common cancer in women worldwide [1]. When detected early, the chance of long-term survival can be as high as 90% due to massive improvements in therapeutic options over the past decades [2,3]. A substantial proportion of patients with breast cancer still receive chemotherapy as part of their treatment, which, however, has detrimental side effects [4,5]. Among the most common toxicities are nausea, emesis, premature menopause and hair loss [6]. Although chemotherapy-induced alopecia is not a life-threatening side effect, it ranks amongst the most troublesome side effects concerning the patients’ quality of life and body image [6,7]. Due to the high chance of a cure by improved systemic therapy, quality of life (QoL) is becoming an increasingly important aspect. Chemotherapy-induced alopecia also affects psychological well-being and has been associated with depression [7,8].

Currently, the most promising method to prevent chemotherapy-induced alopecia is scalp cooling (SC). To date, various studies have reported its efficacy in small patient populations [6,7,10]. SC leads to vasoconstriction, which inhibits cellular drug uptake [6]; furthermore, hypothermia reduces the metabolic rate of hair follicles, finally lowering susceptibility to chemotherapy damage. Concerns that SC might increase scalp metastases have limited its clinical use in the past. However, a recent meta-analysis by Rugo et al. showed no association of scalp metastases with SC [11].

Initial studies on SC were conducted primarily with taxane- and only rarely anthracycline-based chemotherapy [9]. A high efficacy of SC was previously demonstrated for chemotherapy containing taxanes only; however, this does not reflect clinical reality considering that most chemotherapy regimens contain two or more cytostatic agents [9,[12], [13], [14]].

This prospective study aims to evaluate the efficacy of SC in different chemotherapy regimens and hair recovery in the follow-up period.

## 2 Patients and methods

### 2.1 Study design

This study is a prospective interventional single-centre study on women with breast cancer who underwent chemotherapy at the Department of Gynaecology and Obstetrics, Medical University of Innsbruck between May 2018 and February 2021. The local ethics committee approved the study. Written informed consent was obtained from all individual participants included in the study. The clinical trial registration number is NCT04117815.

### 2.2 Patients

The study population included 128 patients; 88 individuals were assigned to the intervention group (CAP) and underwent SC, and 40 patients were allocated to the control group (NCAP). The control group consisted of patients who were eligible to participate in the study and consented for data collection but declined to undergo scalp cooling. Baseline clinical and sociodemographic data were collected. Breast cancer patients undergoing neoadjuvant, adjuvant or palliative chemotherapy were eligible. Chemotherapy consisted of at least four cycles of a taxane- or anthracycline-based regimen, and up to two lines of chemotherapy were allowed. Patients had to be older than 18 years of age. Written informed consent was obtained from all subjects. Exclusion criteria were migraine, Raynaud syndrome or cold allergy, haematological malignancies, scalp metastases, preexisting alopecia, overt cognitive impairment, and insufficient knowledge of the German language.

### 2.3 Scalp cooling procedures

Scalp cooling was performed using the Orbis Paxman Hair Loss Prevention Scalp Cooling System (model: 447 CE). SC treatment was carried out by specially trained staff and was initiated at least 30 min prior to intravenous administration of chemotherapy. The cooling cap was fitted on the patients’ heads as described in the product information recommendations. A temperature between 16 °C and 20 °C was maintained on the scalp of the participants. Cooling was maintained throughout the administration of chemotherapy and was stopped 60–90 min after termination of infusion of cytotoxic agents.

### 2.4 Study endpoints

The primary endpoint was hair preservation (HP), defined as grade 0 or 1 alopecia. The evaluation was conducted by the patients themselves as well as by an expert committee.

The secondary endpoint included patient satisfaction and Quality of life (Qol).

The third endpoint was hair regrowth (HR) determined by medical experts three and six to nine months after the completion of chemotherapy cycles. Adverse events were captured using the case report form.

### 2.5 Assessments of hair loss

To evaluate the state of the patients' hair precisely, five standardized photographs were taken by a professional photographer at five time points during the study: before the start of treatment (T0), at mid-treatment (after half the number of planned cycles (T1)), at the time of the last chemotherapy cycle (T2), 3 months after completion of treatment (T3) and 6–9 months after treatment completion (T4). Patients discontinuing scalp cooling or chemotherapy for any reason were still assessed as planned until T4. Hair loss grading was carried out by photographing the participants’ scalps in both groups. The photo evaluation was blinded and performed by three health care experts using the CTCAEv.4.0 grading system [15]. Grade 0 was defined as no hair loss, grade 1 was defined as hair loss up to 50%, and grade 2 was defined as hair loss of 50% or more. If patients requested discontinuation of SC due to hair loss, this was documented as grade 2 alopecia.

For the purpose of achieving a blinded evaluation, the faces of the patients in the photographs were blacked out to eliminate any kind of bias regarding factors such as age, ethnicity, and type of chemotherapy regimen.

At time points T3 and T4, regrowth after hair loss was also analysed. Currently, there is no standardized protocol or established grading system for the assessment of regrowth after chemotherapy-induced alopecia. Therefore, hair regrowth was assessed according to the definition of therapeutic regrowth as used in dermatology described by Lee et al. [16]: Grade 1, which is any regrowth (<10%), grade 2, which is minor regrowth (10–59%), grade 3, which is major regrowth (60–89%) and grade 4, which is complete regrowth (90–100%).

The patient-reported outcomes included a questionnaire battery composed of the NCI-PRO-CTCAE-ITEMS (National Cancer Institute Patient reported-outcomes Common Terminology Criteria for Adverse Events), EORTC QLQ-C30 (European Organisation for Research and Treatment of Cancer Quality of life questionnaire), BR23update, Body Image Scale, Pro-CTCAEs (Patient reported-outcomes Common Terminology Criteria for Adverse Events) and Hairdex questionnaire. QoL assessment will be reported separately.

### 2.6 Statistical methods/analysis

All analyses were performed using SPSS version 25. The study group was compared with the reference group regarding alopecia and quality of life outcome using linear mixed models with adjustment for age, chemotherapy regimen, tumour type, and baseline body image. Variance estimates were adjusted for the correlation of repeated observations from the same patient. For comparisons between the two groups, a planned sample size of 88 patients in the study group and 40 patients in the reference group was sufficient to detect an effect size of d = 0.55 in a two-sided *t*-test (alpha = 0.05, beta = 0.20). As the statistical power of an analysis by means of linear mixed models usually exceeds that of the *t*-test, it is expected that even somewhat smaller effect sizes can be detected in the planned analysis. Group (study group and reference population group) and time point (T0-T4) were included as main effects to investigate changes over time and group differences. Group-by-time interactions indicated different courses of alopecia in the two groups. For baseline characteristics the p value was calculated using Mann-Whitney-U-Test, Chi2-Test and Fishers’ Exact test.

## 3 Results

### 3.1 Study population

**Fig. 1:**
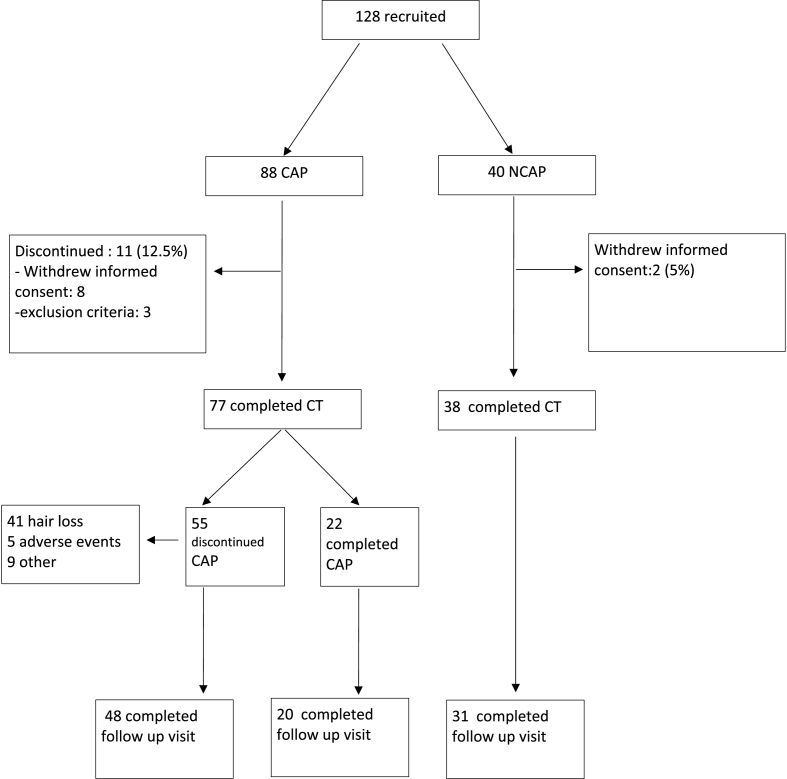
Flowchart. CAP = intervention group; CT = chemotherapy; NCAP = control group.

The study population included 128 patients. The distribution of patients is shown in Fig. 1. The baseline characteristics of our patients are shown in Table 1 and were well balanced in regard to age, menopausal status and tumour features. The median age in the CAP group was 51 (23–81) and 54.5 (32–80) in the NCAP group. Eighty-eight patients who met the inclusion criteria were assigned to the intervention group (CAP) and underwent SC. The other 40 patients were allocated to the control group (NCAP). In the CAP group, 11 patients did not complete the study, which was a dropout rate of 12.5%. Eight (of 11) patients discontinued the trial after declining further application of SC, and three (of 11) patients dropped out due to exclusion criteria that were not apparent during screening. In the control group, the dropout rate was 5%, as two patients withdrew their informed consent during the study. Follow-up was completed for 88.3% of the patients in the intervention group and for 81.6% in the control group.

**Table 1:** Patient characteristics.

	CAP	NCAP	ALL	p value
	n = 77 (%)	n = 38 (%)	n = 115 (%)	
**age (ys): median (range)**	51 (23–81)	54.5 (32–80)	53 (23–81)	0.115^1^
**menopausal status**				0.321^2^
premenopausal	40 (51.9)	16 (42.1)	56 (48.7)	
postmenopausal	37 (48.1)	22 (57.9)	59 (51.3)	
**setting CT**				0.071^2^
neoadjuvant	51 (66.2)	26 (68.4)	77 (67)	
adjuvant	20 (26)	9 (23.7)	29 (25.2)	
palliativ	6 (7.8)	3 (7.9)	9 (7.8)	
**tumour features**				
**subtype**				0.661^3^
invasive ductal	69 (89.6)	36 (94.7)	105 (91.3)	
invasive lobular	4 (5.2)	1 (2.6)	5 (4.35)	
other	4 (5.2)	1 (2.6)	5 (4.35)	
**grading**				0.03^2^
1	6 (7.8)	1 (2.6)	7 (6.1)	
2–3	71 (92.2)	37 (97.4)	108 (93.9)	
**estrogen receptor**				0.315^2^
positive	50 (64.9)	21 (55.3)	71 (61.7)	
negative	27 (35.1)	17 (44.7)	44 (38.3)	
**progesterone receptor**				0.553^2^
positive	47 (61)	21 (55.3)	68 (59.1)	
negative	30 (39)	17 (44.7)	47 (40.9)	
**HER2-status**				0.160^2^
positive	27 (35.1)	8 (21.1)	35 (30.4)	
negative	48 (62.3)	30 (78.9)	78 (67.8)	
unknown	2 (2.6)	0	2 (1.7)	
**Ki 67 status**				0.879^2^
<20%	17 (22)	10 (26.3)	27 (23.5)	
>20%	58 (75.3)	27 (71)	85 (73.9)	
unknown	2 (2.7)	1 (2.6)	3 (2.6)	
**tumour size**				0.997^2^
pT1	35 (45.5)	17 (44.7)	52 (45.2)	
pT 2-3	40 (51.9)	20 (52.7)	60 (52.2)	
pT unknown	2 (2.6)	1 (2.6)	3 (2.6)	

^1^ = Mann-Whitney-U-Test; ^2^ = Chi^2^-Test; ^3^ = Fishers' Exact test.

CAP = intervention group; CT = chemotherapy; HER2 = human epidermal growth factor receptor 2; NCAP = control group; ys = years.

### 3.2 Hair loss

**Fig. 2:**
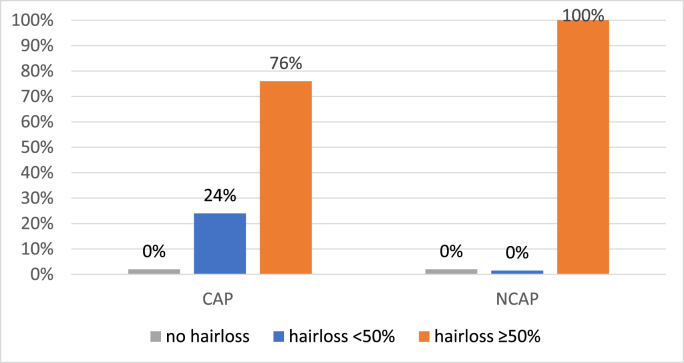
Hairloss patients‘ self-evaluation CAP vs NCAP N = 113. CAP = intervention group; NCAP = control group.

Alopecia was evaluated by the patients themselves and by an expert group. Twenty-four percent of patients in the CAP group and 0% in the NCAP group evaluated their hair loss as grade 1 (<50% hair loss) (P = 0.001). However, none of the patients graded their hair loss as grade 0 (Fig. 2).

**Fig. 3:**
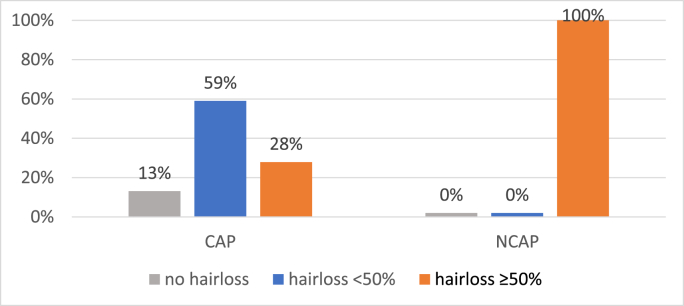
Hairloss experts’ evaluation median CAP vs NCAP N = 93. CAP = intervention group; NCAP = control group.

Experts evaluated the hair loss in CAP group members as grade 0 in 13%, grade 1 in 59% and grade 2 in 28%. This showed hair preservation in 72% of patients using SC, whereas hair preservation was 0% in the NCAP group (P ≤ 0.001) (Fig. 3).

**Fig. 4:**
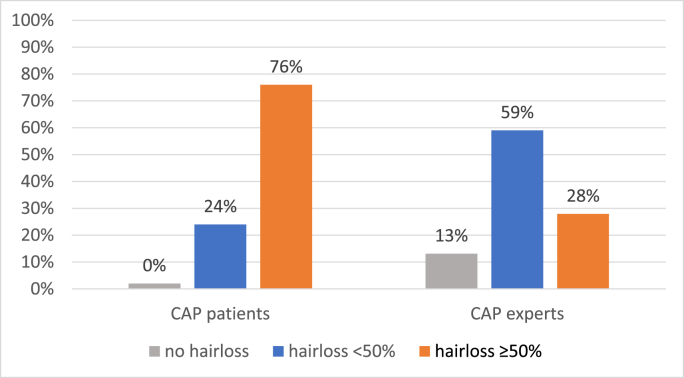
CTCAE patients vs experts CAP. CAP = intervention group; Exp = experts‘ evaluation; NCAP = control group; Pat = patients’ self-evaluation.

Patients as well as experts agreed on the evaluation in the NCAP group (100% alopecia). Interestingly, a significant difference was noted between evaluation by experts (72%, <50% hair loss) and patients’ self-assessment (24%, <50% hair loss) regarding HP in the CAP group (Fig. 4).

### 3.3 Effect of SC-specific chemotherapy regimens

SC was more effective in preventing alopecia in patients receiving taxane monotherapy than in patients receiving an anthracycline-taxane-based regimen. Using the patients’ evaluation, 50% of patients with taxane monotherapy had grade 1 alopecia compared to 17% of patients with anthracycline-taxane CT (50% vs. 17.2%, P = 0.018). Experts evaluated 17% of patients with taxane monotherapy as grade 0 and 42% as grade 1. In comparison, the experts evaluated the hair loss of patients with an anthracycline-taxane CT as grade 0 in 7.9% and grade 1 in 49% (P = 0.061).

Regarding the sequence of CT (taxanes followed by anthracyclines or vice versa), there was no significant difference in hair loss. SC was successful in 39% of patients who received taxanes first and in 20% of patients who received anthracyclines first (Table 2).

**Table 2:** Influence of CT regimen on alopecia patients’ evaluation.

	Patients	Grade:0–1 alopecia (%)	Grade:2 alopecia (%)	Grade alopecia unknown (%)
**CAP**	(N = 77)			
TX Mono	12	6 (50)	5 (41.7)	1 (8.3)
TX and AC	64	11 (17.2)	52 (81.2)	1 (1.6)
sequence TX/AC	(N = 64)			
TX followed by AC	13	5 (38.5)	8 (61.5)	0 (0)
AC followed by TX	25	5 (20)	20 (80)	0 (0)
TX AC simultaneously	26	1 (3.85)	24 (92.3)	1 (3.85)
other	1	1 (100)	0 (0)	0 (0)
**NCAP**	(N = 38)			
TX Mono	6	0 (0)	6 (100)	0 (0)
TX and AC	32	0 (0)	32 (100)	0 (0)

AC = anthracycline; CAP = intervention group; Mono = monotherapy; NCAP = control group; TX = taxane.

If patients requested discontinuation of SC due to hair loss, this was documented as grade 2 alopecia.

### 3.4 Discontinuation of SC

Seventy-one percent (n = 55) of patients in the CAP cohort did not use SC until the end of their CT. Reasons for discontinuation of SC were summarized in three different scenarios: first, hair loss (75%); second, discontinuation for logistical reasons (16%); and third, the patient not tolerating SC (9%). Sixteen percent (n = 9) of patients stopped using SC for reasons unrelated to adverse events of SC or hair loss (e.g., transfer of care, discontinuation of CT due to adverse events or disease progression). Nine percent of patients reported slight headaches, scalp pain and a generalized feeling of cold.

### 3.5 Patient satisfaction and impact of scalp cooling on quality of life (QoL)

We did not observe a superiority of scalp cooling in terms of QoL outcome (Fig. 6). This may be a result of a high discontinuation rate. More than two-thirds of patients reported alopecia as the primary reason of an early discontinuation of scalp cooling. Based on patient-reports, the application of scalp cooling in routine care for all chemotherapy regimens hence seems to be limited.

**Fig. 5:**
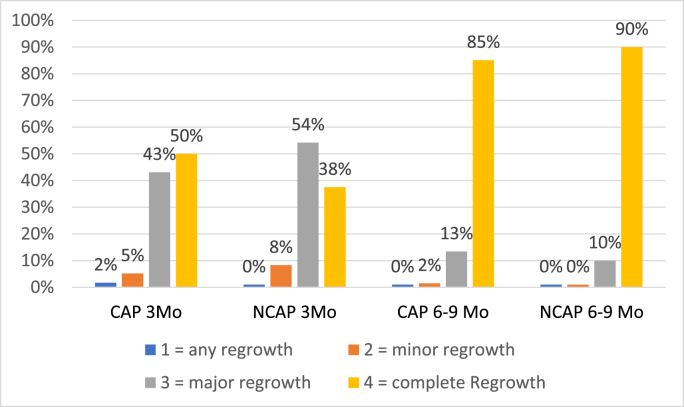
Regrowth 3 and 6–9 months CAP vs NCAP. CAP = intervention group; NCAP = control group.

**Fig. 6:**
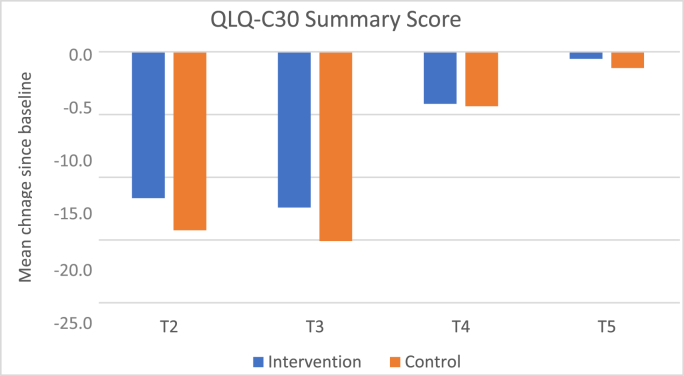
Change of quality of life (EORTC QLQ-C30 sum score and Body Image Scale) from baseline to follow-up assessment.

### 3.6 Hair regrowth

Hair regrowth was evaluated by experts. After three months, 50% of patients in the CAP group and 38% in the NCAP group had complete regrowth of their hair. At the six-to nine-month follow-up, complete regrowth was observed in 85% of patients in the CAP group and 90% of patients in the NCAP group (Fig. 5). Concerning HR three and six to nine months after CT, no significant differences between the two groups were found.

### 3.7 Adverse events

A total of 9.1% (n = 5) of the patients stopped using SC due to experiencing adverse events such as scalp pain, extreme sensation of cold or headaches.

## 4 Discussion

SC is currently the most promising approach to prevent chemotherapy-induced alopecia. The Hair-Safe study is among the first studies evaluating hair loss not only by health care experts but also by patients themselves. The assessments were not combined but examined separately. In addition, hair regrowth after treatment was assessed. Coolbrandt et al. also published self-reported outcomes for approximately 82% of their breast cancer patients at the end of SC and one year after treatment [17]. In our study, hair preservation was significantly higher in the group undergoing SC than in the control group when using patient and expert evaluations. Previous studies have already shown the high efficacy and safety of this application [7,10]. Interestingly, varying success rates of SC between 30 and 80% have been described [6,9,10,18,19]. Discrepancies in success rates may be attributed to different trial designs and patient populations, CT regimens, timeframes for evaluating hair loss and types of devices used. In the review by Wang et al. [20], the results of 3 different devices were presented, and the effectiveness of SC therapy was dependent on different cooling equipment. The success rate was between 55 and 75% depending on the device.

Remarkably, our study shows a significantly worse evaluation of the CAP group regarding patient evaluation in comparison to expert evaluation. In fact, none of the patients in the CAP group described their hair loss as grade 0 alopecia, whereas 13.1% were evaluated as grade 0 by experts. In the NCAP group, there was no difference between the evaluations because both patients and experts assessed 100% of the participants as having grade 2 alopecia (≥50% hair loss). A possible explanation could be that patients themselves seem to be more critical in evaluating their hair loss. This underlines the importance of patient involvement in reported outcomes, demonstrating that minor hair loss should be considered normal in successful SC. It is possible that the first instance of minor hair loss can subjectively be perceived as grade 2 alopecia by patients due to preconceived expectations or other psychological aspects. This could explain the phenomenon of patients perceiving their hair loss as grade 2, whereas experts evaluated it as grade 1 or even grade 0.

The efficacy of SC is influenced by many factors, such as age, infusion time, ethnic differences in head shape and scalp temperature [21,22]. However, the most important influence is the chemotherapeutic agent. In accordance with previously published studies [9,[12], [13], [14]], we observed that SC was more effective in patients receiving taxane monotherapy than in patients receiving an anthracycline-taxane-based regimen. Using the patients’ evaluation, 50% of patients with taxane monotherapy had grade 1 alopecia compared to 17.2% of patients with an anthracycline-taxane-based regimen. Initial studies on SC mainly included patients undergoing taxane monotherapy [18,23,24]. These studies demonstrated fairly good efficacy but do not fully depict the clinical reality where patients usually receive CT regimens containing taxanes as well as anthracyclines.

In our study, patients' and experts’ opinions matched in their evaluation of the hair preservation rate concerning taxane monotherapy. Interestingly, the evaluation differed regarding patients receiving anthracycline-taxane-based CT. Almost one-fifth (17%) of patients undergoing anthracycline-taxane-based CT evaluated their hair loss as grade 1 compared to more than half (57%) of experts evaluating hair loss as grade 1 or below. The subjective patient self-evaluation may be influenced by the medical education on SC that patients received, knowing that hair loss was more likely to happen in an anthracycline-taxane-based regimen. The primary reason for discontinuation of SC was hair loss, which is consistent with other studies where hair loss was also the main reason for SC discontinuation [7,12,25].

Regarding the CT regimen sequence, there was no significant difference in hair loss in our study. Bajpai et al. indicated in their study that in patients receiving taxanes followed by anthracyclines, SC procedures were more effective in comparison to those receiving anthracyclines followed by taxanes [6]. It is noteworthy that the CAP group in the study of Bajpai et al. only contained half the number of patients compared to the present study.

This study additionally examined the potentially positive effect of SC on regrowth after chemotherapy-induced alopecia. To date, only a few studies have observed hair regrowth after CT [6,7,10,12]. Although the exact mechanism by which SC prevents alopecia is not yet fully understood, SC presumably leads to protection of the hair follicle. In most individuals, chemotherapy-induced alopecia is a reversible process [26]. Therefore, a potential impact of SC in this time frame may lead to faster or more voluminous regrowth. Hair regrowth after chemotherapy in our study showed no significant differences between the CAP and NCAP groups 3 months after CT or 6–9 months after CT. Our results are consistent with the findings of the Coolhair study by Smetanay et al. which showed no significant difference between the CAP and NCAP groups 3 and 6 months after CT regarding expert evaluation [7]. In contrast, Kinoshita et al. reported a significant difference between the CAP and NCAP groups regarding patients with an increase of at least 50% of hair after 12 weeks. Regrowth was judged by 2 independent assessors and the patient. In addition, the proportion of patients who recovered from grade 2 to grade 0 alopecia over 12 weeks after CT was significantly higher in the CAP group [10].

Likewise, data published by Ohsumi et al. also report significantly better regrowth in their CAP vs. NCAP groups. Hair regrowth was judged at five time points by two experts using a 4-step score and by patients using questionnaires. They found a significant difference regarding expert evaluation at all time points and at four and seven months after CT using patients’ questionnaires [12]. Furthermore, Bajpai et al. reported significantly better regrowth in patients who underwent SC after chemotherapy-induced alopecia than in patients who did not undergo SC [6].

All the abovementioned studies used different time frames and scores for the evaluation of regrowth. We used a well-established dermatology scale, and the evaluation was performed by three experts in the field with substantial clinical experience. Therefore, comparison of studies regarding regrowth after chemotherapy-induced alopecia is currently difficult due to lack of standardized and validated scales and widely differing assessment time points. Eventually, the use of SC mediates accelerated early regrowth, as the work of Kinoshita et al. suggests, but lacks maintenance of this benefit over long-term observation. Additionally, it remains unclear whether there is a beneficial effect of SC on the prevention of permanent alopecia. However, despite the long follow-up in our study, no cases of permanent alopecia were noted.

SC is a safe technique with no serious safety concerns. Our study confirms the results of previous studies in regard to the high efficacy and safety of this application [6,9,11,12,18,19,21,22].

A major strength of our study is the separate examination of expert and patient evaluations. In addition, our study examines one of the largest patient populations regarding SC and especially regrowth after SC. Additionally, the Hair-Safe study has the longest follow-up period compared to previous studies.

We report a dropout rate of 13% in the CAP group and 5% in the NCAP group. However, our study took place during the COVID-19 pandemic with the necessity of postponing follow-up appointments. This especially affected the first 3 months after CT follow-up. One of the limitations of our study is that patients were not randomized. Additionally, we did not adjust for confounders that may have potentially influenced the study's results. Our study is a single-centre study and included patients with 14 different CT regimens. We acknowledge that due to our sample size, it was impossible to evaluate the impact of each regimen on the efficacy of SC individually. To explore this question, multicentre studies with larger patient populations are needed.

## 5 Conclusion

One key finding of our study is the importance of extensive patient education, especially emphasizing that minor hair loss may occur even during successful SC. Overall, SC is the most promising approach to prevent alopecia in terms of efficacy and safety. Patients can highly benefit from the application of scalp cooling devices. SC should be integrated into clinical practice and targeted towards patients with potentially high success rates of SC selected by chemotherapy regimen. Ideally, health insurance should cover the costs of treatment, and it should be made broadly available.

## Declaration of competing interest

The authors declare no conflict of interest.
